# Prediction model for potential depression using sex and age-reflected quantitative EEG biomarkers

**DOI:** 10.3389/fpsyt.2022.913890

**Published:** 2022-09-07

**Authors:** Taehyoung Kim, Ukeob Park, Seung Wan Kang

**Affiliations:** ^1^iMediSync Inc., Seoul, South Korea; ^2^National Standard Reference Data Center for Korean EEG, Seoul National University College of Nursing, Seoul, South Korea

**Keywords:** depression, EEG, classification, biomarker, prediction, machine learning

## Abstract

Depression is a prevalent mental disorder in modern society, causing many people to suffer or even commit suicide. Psychiatrists and psychologists typically diagnose depression using representative tests, such as the Beck’s Depression Inventory (BDI) and the Hamilton Depression Rating Scale (HDRS), in conjunction with patient consultations. Traditional tests, however, are time-consuming, can be trained on patients, and entailed a lot of clinician subjectivity. In the present study, we trained the machine learning models using sex and age-reflected z-score values of quantitative EEG (QEEG) indicators based on data from the National Standard Reference Data Center for Korean EEG, with 116 potential depression subjects and 80 healthy controls. The classification model has distinguished potential depression groups and normal groups, with a test accuracy of up to 92.31% and a 10-cross-validation loss of 0.13. This performance proposes a model with *z*-score QEEG metrics, considering sex and age as objective and reliable biomarkers for early screening for the potential depression.

## Introduction

Depression, a major cause of global burden, can be a life-threatening mental disorder (1). Depression is related to sadness or bereavement, but it can persist even after the external causes of these emotions were resolved. There are even some patients with a severe state of depression who have no external causes (2). The main symptoms of depression were known as sadness, crying, lack of energy, difficulties in decision making, and so on (3). Rapid increases in patients with potential depression, in consequence of post-COVID19 syndrome and economic turndown, have raised serious societal concerns (4). The World Health Organization (WHO) reported that more than 300 million people worldwide suffer from depression. The bigger problem is that the procedure of depression diagnosis is complicated. Diagnosis of depression is usually done through interviews with physicians and accompanying tests, such as Beck’s depression inventory (BDI) or Hamilton Depression Rating Scale (HDRS). However, this process is time-consuming and burdensome for the patient. In addition, it is difficult to take quick measures due to the shortage of professionals in hospitals and counseling facilities and to make accurate self-diagnosis because of ambiguities in the symptoms. This can lead patients to have a significant progression of symptoms before visiting the clinic.

Recently, several studies are trying to find biomarkers for depression using brain activity to diagnose in a more objective and time-saving way (5, 6). Among the methods of measuring brain activity, non-invasive EEG is best-suited as a quick and simple way to diagnose depression. There are lots of advantages in using the EEG rather than other brain measuring methods: less time-consuming, cost-efficient, easy to measure, and convenient. The most representative indicator in the EEG signal is Band power: Delta (1–4 Hz), Theta (4–8 Hz), Alpha (8–12 Hz), Beta (12–30 Hz), and Gamma (30–45 Hz). Prior studies showed that 25% of studies used band power for the biomarkers of depression (1). Especially, Alpha band power is accounted for a large portion for an important feature among them (7–10).

However, QEEG is user-independent data, which has lots of variability. Our previous study reported the sex- and age-differentiated standardized quantitative EEG (QEEG) normative database (ISB-NormDB), which can remove user-independent variability (11). Through this database and sex- and age-fitted model, band power data can be converted to sex- and age-matched standardized band power values (Z-scored band power). These standardized band power can be a major candidate for biomarkers for the EEG-based prediction model. There were several previous studies that improve the model performance using this gender and age-matched standardized features (12, 13).

Predicting result of disease diagnosis using a classification model is an example of how to discover influential biomarkers. The more influential biomarkers are, the greater the performance of the model. There are also studies that tried to detect depression using artificial neural network (14, 15). However, the disadvantage is that it is difficult to clearly know which biomarker contributes greatly when using a neural network-based model. In addition, many previous studies have a limitation that the number of subjects were not that many (under 15 per group), and all subjects were already clinically diagnosed with Major depressive disorder (MDD).

The objective of the present study is to build an early screening model for potential depression using sex- and age-matched QEEG features. To satisfy this, we divided potentially depressed people and healthy people from our database by optimal BDI criteria (16). We extracted features that contributed greatly to depression prediction in statistical and deductive ways, and we built the early screening model based on those extracted features. The significance of our study lies in predicting potential depression among those who have not been clinically diagnosed. The significance of our study is that it contributes to diagnosing depression in the early stage in a fast and low-cost way, assisting the doctor’s clinical decision and helping potential depression of the patient.

## Materials and methods

### Data

All data were obtained from the National Standard Reference Data Center for Korean EEG. The data center has approximately 1,300 standard QEEG data (called ISB-NormDB). The experimental procedure for data was approved by the Research Ethics Committee of the Seoul National University and informed consent was signed by each participant prior to the recording.

### Subjects

Based on the BDI cut-off criteria (14), a total of 196 subjects were selected, including 116 subjects with the potential depression (men = 23, women = 95, age = 58.66 ± 15.08 years, BDI = 21.17 ± 6.28) and 80 healthy controls (men = 44, women = 36, age = 48.66 ± 16.71 years, BDI = 0) from the National Standard Reference Data Center for Korean EEG (BDI cut-off: 14.48). All subjects did not take medicine, had never been diagnosed with mental illness, and had never visited a hospital for depression. Hereafter, the group of 116 depression subjects is denoted as a potential depression group and the group of 80 healthy controls as a normal group, respectively.

### EEG recordings

**FIGURE 1:**
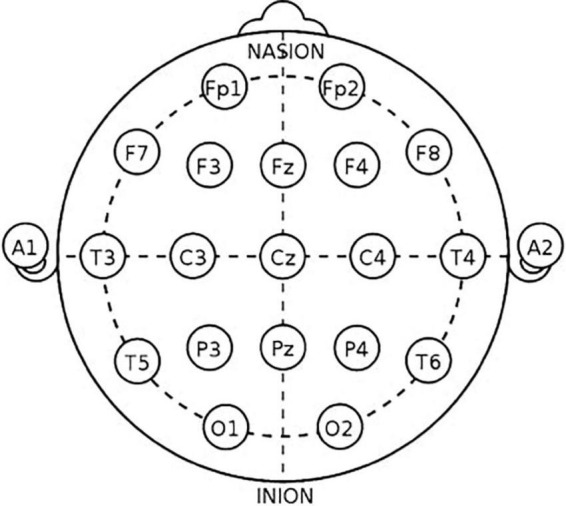
Montage of the international 10–20 system.

Electroencephalogram were recorded from 19 active wet electrodes (FP1, FP2, F7, F3, Fz, F4, F8, T3, C3, Cz, C4, T5, P3, Pz, P4, T6, O1, and O2), using the international 10–20 system (Mitsar, Inc., Russia, Petersburg) (Figure 1). The sampling rate was 250 Hz. The ground and reference electrodes were attached to the left and right earlobe, respectively. The contact impedance was kept below 10 kΩ. During the recording, participants were relaxed and awake with their eyes closed. The data were recorded for at least 5 min.

## EEG analysis

### Pre-processing

Overall EEG pre-processing was basically performed using denoising algorithm in iSyncBrain (iMediSync, Inc., Korea).^1^ The raw EEG data was filtered with notch filter. Low cut-off and high cut-off frequencies were 1 and 45 Hz, respectively. Re-referencing was performed using Common average reference (CAR). Artifacts were removed by bad epoch rejection and Independent Component Analysis (ICA)-based algorithm.

### Group analysis

To find feature candidates for classification model, we compared absolute band power and relative band power between potential depression group and normal group. Band power extractions and statistical test were performed on iSyncBrain. The topographical mapping (topomap) images were also generated by iSyncBrain. We analyzed eight frequency band power: Delta (1–4 Hz), Theta (4–8 Hz), Alpha1 (8–10 Hz), Alpha2 (10–12 Hz), Beta1 (12–15 Hz), Beta2 (15–20 Hz), Beta3 (20–30 Hz), and Gamma (30–45 Hz). Alpha bands and beta bands were divided for more granular frequency analysis (17, 18). Absolute power and relative power between the group for each frequency band were compared.

Absolute band power is a spectral band power based on fast Fourier transform (FFT) provided by iSyncBrain.

Relative band power is the absolute power in a specific frequency band divided by the total power. We first performed the Shapiro-Wilks test or the Kolmogorov-Smirnov test for normality, and then performed the independent *T*-test or the Mann-Whitney *U*-test to test a significant difference in the band power between groups for each frequency band.

### Sex and age-matched features

ISB-NormDB is sex- and age-differentiated standardized QEEG normative database (11). ISB-NormDB has total 1,289 subjects’ QEEG data (553 men, 736 women, ages from 4.5 to 81 years). In previous study, they verified that QEEG feature varies with age and gender that constructed standardized models with rigor criteria for each age and gender. A raw feature, such as Absolute band power and Relative band power, can be converted to z-scored values by this NormDB model. The converted value can represent how much the raw feature has bigger or less than standard people of the same age and gender (call these converted features “Z-scored” features). Therefore, the effects of gender and age can be corrected by using z-scored features.

### Feature extraction and selection

Four distinctive features were obtained from band powers: Absolute band power, Relative band power, Absolute z-scored band power, and Relative z-scored band power. Gamma band (30–45 Hz) was excluded from the analysis because the gain of overall feature importance was obtained when it was removed. To remove the differences in sex and age between groups, we matched each subject’s sex and age to data in the National Standard Reference Data Center for Korean EEG and calculated z-scored band power, which are Absolute z-scored band power and Relative z-scored band power. A total of 532 features (4 kinds × 19 channels × 7 bands) were extracted for candidates for the final feature.

**FIGURE 2:**
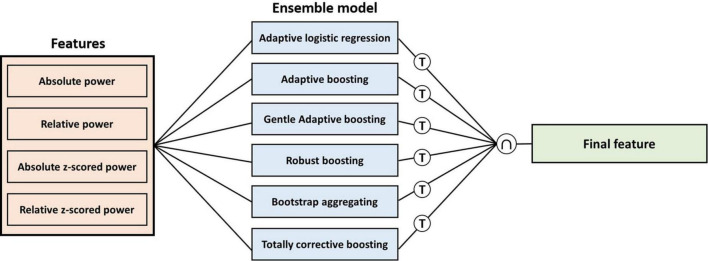
Procedure of calculating feature importance for each feature in each ensemble model. T means threshold of the number of the highest score features for each model, and ∩ means intersection for the highest feature in each model.

We computed feature importance by summing changes in the mean squared error due to splits on every feature and dividing the sum by the number of branch nodes in tree-based ensemble models to select the final feature. A total of six tree-based ensemble model were used to compute feature importance: Adaptive logistic regression, Adaptive boosting, Gentle adaptive boosting, Robust boosting, Bootstrap aggregating, and Totally corrective boosting. Once the feature importance has been calculated in each model, we adopt an intersection of features with higher scores in each model as the final feature (Figure 2).

### Model training

In model training, 80% of the total data was used for training and 20% for testing. For intermediate verification, a 10-fold validation model using a training set was built separately. Data was shuffled before the training. We compared the performance of 10 classification models: Logit boost (LB), Error-correcting output codes (ECOC), Discriminant analysis (DA), Support vector machine (SVM), Gaussian kernel (GK), K-nearest neighbor (KNN), regularized SVM (rSVM), Naïve bayes (NB), Decision tree (DT), and AdaboostM1 (AdaM1), adjusting the number of features based on feature importance scores.

## Results

### Group analysis

**FIGURE 3:**
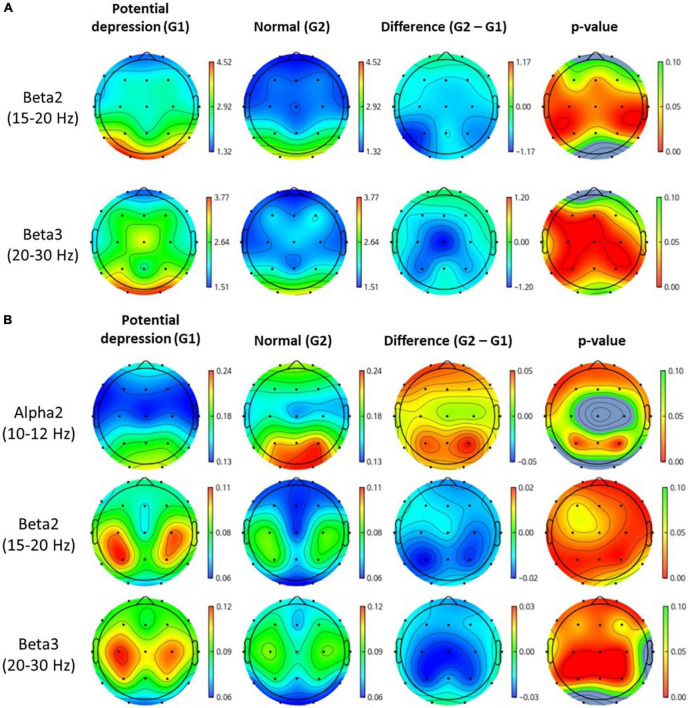
Topomap of frequency bands that have significant difference between groups. Unit of spectral power is μV^2^. **(A,B)** Represent absolute power and relative power of each group, respectively.

Figure 3 shows topomap of frequency bands that have significant difference between groups. The spectral power of each group was average value of subjects in each group. Potential depression group had significantly larger power in beta2 and beta3 both in absolute band power and relative band power than normal group (*p* < 0.05). However, potential depression group had significantly lower relative band power in alpha2 (*p* < 0.05). Beta2 and beta3 showed significant differences in almost all areas in the brain, while alpha2 showed significant differences mainly in frontal, temporal, and parietal domains. Tables 1, 2 illustrate significant absolute band power and relative band power, respectively. Statistical analysis for each electrode was made and significances were marked as star. In absolute band power, almost all electrodes showed significance, except for pre-frontal and occipital areas. In relative band power, almost all electrodes showed significance except for occipital area.

**TABLE 1:** Numerical values for each channel for absolute beta2 and beta3 power.

Channels	Abs beta2			Abs beta3		
		
	Normal	Depression	Significance	Normal	Depression	Significance
Fp1	1.31 ± 1.023	1.67 ± 1.804		1.53 ± 1.211	1.80 ± 1.251	
Fp2	1.32 ± 1.023	1.69 ± 1.925		1.5 ± 1.0210	1.77 ± 1.232	
F7	1.43 ± 1.019	1.97 ± 1.945	*	1.63 ± 0.958	2.18 ± 1.488	*
F3	1.66 ± 1.266	2.21 ± 2.564		1.97 ± 1.311	2.57 ± 2.127	*
Fz	1.59 ± 1.150	2.25 ± 2.443	*	1.79 ± 1.295	2.47 ± 2.021	*
F4	1.72 ± 1.279	2.33 ± 2.629		2.08 ± 1.467	2.60 ± 1.985	*
F8	1.51 ± 1.147	1.96 ± 2.149		1.72 ± 1.118	2.08 ± 1.526	
T3	1.60 ± 1.240	2.10 ± 1.820	*	1.64 ± 1.367	2.00 ± 1.435	
C3	1.66 ± 1.327	2.33 ± 2.203	*	1.73 ± 1.219	2.52 ± 2.021	*
Cz	1.59 ± 1.202	2.30 ± 2.578	*	1.98 ± 1.402	3.19 ± 3.151	*
C4	1.72 ± 1.417	2.47 ± 2.309	*	1.78 ± 1.251	2.48 ± 1.902	*
T4	1.54 ± 1.303	2.11 ± 1.830	*	1.64 ± 1.324	2.01 ± 1.407	
T5	2.77 ± 2.151	3.95 ± 3.700	*	2.27 ± 1.455	2.95 ± 1.905	*
P3	2.50 ± 2.234	3.50 ± 3.230	*	2.14 ± 1.694	3.09 ± 2.455	*
Pz	1.90 ± 1.648	2.55 ± 2.455	*	1.79 ± 1.299	2.35 ± 1.818	*
P4	2.41 ± 2.129	3.22 ± 2.917	*	2.17 ± 1.633	2.92 ± 2.245	*
T6	2.84 ± 2.276	3.67 ± 3.117	*	2.29 ± 1.441	2.86 ± 1.853	*
O1	3.68 ± 3.526	4.53 ± 4.081		3.12 ± 1.757	3.70 ± 2.348	
O2	3.71 ± 3.105	4.34 ± 3.672		3.19 ± 1.929	3.76 ± 2.431	

Significance was marked as star (p < 0.05). Each numerical values are mean ± standard deviation.

**TABLE 2:** Numerical values for each channel for relative alpha2, beta2, and beta3 power.

Channels	Rel alpha2			Rel beta2			Rel beta3		
			
	Normal	Depression	Significance	Normal	Depression	Significance	Normal	Depression	Significance
Fp1	0.20 ± 0.145	0.15 ± 0.105	*	0.06 ± 0.031	0.07 ± 0.042	*	0.07 ± 0.042	0.09 ± 0.049	*
Fp2	0.20 ± 0.135	0.16 ± 0.114	*	0.05 ± 0.030	0.07 ± 0.044	*	0.07 ± 0.039	0.09 ± 0.049	*
F7	0.17 ± 0.118	0.13 ± 0.090	*	0.06 ± 0.025	0.07 ± 0.032	*	0.07 ± 0.032	0.08 ± 0.042	*
F3	0.16 ± 0.121	0.13 ± 0.101	*	0.06 ± 0.035	0.07 ± 0.043	*	0.08 ± 0.048	0.10 ± 0.059	*
Fz	0.16 ± 0.122	0.13 ± 0.103	*	0.05 ± 0.035	0.07 ± 0.044	*	0.06 ± 0.045	0.08 ± 0.054	*
F4	0.16 ± 0.114	0.13 ± 0.100	*	0.06 ± 0.030	0.07 ± 0.046	*	0.08 ± 0.049	0.09 ± 0.054	*
F8	0.16 ± 0.107	0.13 ± 0.093	*	0.06 ± 0.023	0.07 ± 0.037	*	0.07 ± 0.032	0.09 ± 0.044	*
T3	0.14 ± 0.073	0.12 ± 0.069	*	0.07 ± 0.029	0.08 ± 0.035	*	0.08 ± 0.032	0.09 ± 0.042	*
C3	0.16 ± 0.103	0.14 ± 0.091		0.08 ± 0.045	0.10 ± 0.051	*	0.09 ± 0.047	0.12 ± 0.061	*
Cz	0.14 ± 0.099	0.12 ± 0.095		0.05 ± 0.032	0.07 ± 0.043	*	0.07 ± 0.048	0.10 ± 0.069	*
C4	0.15 ± 0.086	0.13 ± 0.082		0.08 ± 0.043	0.10 ± 0.052	*	0.09 ± 0.046	0.11 ± 0.060	*
T4	0.14 ± 0.077	0.12 ± 0.067	*	0.07 ± 0.028	0.09 ± 0.042	*	0.08 ± 0.034	0.09 ± 0.043	
T5	0.18 ± 0.115	0.16 ± 0.105		0.06 ± 0.032	0.08 ± 0.055	*	0.06 ± 0.034	0.07 ± 0.046	*
P3	0.22 ± 0.132	0.17 ± 0.115	*	0.08 ± 0.048	0.10 ± 0.06	*	0.08 ± 0.049	0.10 ± 0.063	*
Pz	0.21 ± 0.133	0.18 ± 0.118	*	0.06 ± 0.036	0.08 ± 0.047	*	0.07 ± 0.041	0.08 ± 0.050	*
P4	0.23 ± 0.137	0.18 ± 0.115	*	0.08 ± 0.047	0.10 ± 0.052	*	0.08 ± 0.048	0.10 ± 0.058	*
T6	0.19 ± 0.128	0.16 ± 0.122		0.06 ± 0.037	0.07 ± 0.049		0.06 ± 0.036	0.06 ± 0.042	
O1	0.23 ± 0.156	0.20 ± 0.149		0.05 ± 0.030	0.07 ± 0.051	*	0.05 ± 0.038	0.06 ± 0.042	
O2	0.24 ± 0.164	0.21 ± 0.158		0.05 ± 0.032	0.06 ± 0.045		0.05 ± 0.040	0.06 ± 0.044	

Significance was marked as star (p < 0.05). Each numerical values are mean ± standard deviation.

### Classification model performance

The performances of binary classification models according to the number of final features were showed in Table 3. The best classification result was when AdaM1 had 21, 23, and 28 features, respectively, showing 92.31% test accuracy. 10-fold cross validation loss for each result were 0.14, 0.17, and 0.13, respectively. Sensitivity and specificity were 0.88 and 1, respectively. The highest test accuracy and lowest cross-validation loss were obtained when using 28 features of the AdaM1 model. Table 4 shows the information on 28 features used in an AdaM1 model. Out of the 28 features, 15 were selected from the relative z-scored band power. At the frequency band level, beta and alpha bands were the most common, with 11 and 8, respectively, and at the brain level, the frontal and temporal areas were the most common with 8.

**TABLE 3:** Comparison of each model performance according to number of final features.

Number of features	LB	ECOC	DA	SVM	GK	KNN	rSVM	NB	DT	AdaM1
3	56.41	58.97	58.97	58.97	53.85	53.85	58.97	61.54	56.41	61.54
4	56.41	64.10	61.54	64.10	48.72	56.41	64.10	61.54	71.79	66.67
7	53.85	64.10	64.10	64.10	58.97	64.10	64.10	66.67	66.67	64.10
8	64.10	69.23	61.54	69.23	53.85	64.10	69.23	69.23	56.41	61.54
10	66.67	66.67	64.10	66.67	56.41	71.79	66.67	69.23	53.85	71.79
11	69.23	64.10	64.10	64.10	64.10	69.23	64.10	71.79	48.72	74.36
12	66.67	64.10	69.23	64.10	58.97	69.23	64.10	69.23	58.97	76.92
13	71.79	69.23	74.36	69.23	56.41	66.67	69.23	74.36	64.10	74.36
14	71.79	66.67	71.79	66.67	69.23	66.67	64.10	69.23	51.28	79.49
15	79.49	66.67	71.79	66.67	58.97	66.67	66.67	71.79	58.97	74.36
16	76.92	66.67	66.67	66.67	53.85	74.36	66.67	64.10	79.49	74.36
17	82.05	66.67	64.10	66.67	53.85	74.36	66.67	61.54	76.92	79.49
20	76.92	61.54	61.54	61.54	46.15	66.67	58.97	66.67	74.36	82.05
**21**	**87.18**	**66.67**	**71.79**	**66.67**	**61.54**	**69.23**	**66.67**	**66.67**	**74.36**	**92.31**
22	82.05	69.23	71.79	69.23	58.97	74.36	69.23	66.67	71.79	87.18
**23**	**82.05**	**69.23**	**71.79**	**69.23**	**56.41**	**74.36**	**69.23**	**66.67**	**76.92**	**92.31**
24	84.62	66.67	71.79	66.67	58.97	74.36	69.23	64.10	71.79	87.18
26	89.74	66.67	69.23	66.67	56.41	61.54	69.23	66.67	69.23	84.62
27	82.05	61.54	69.23	61.54	53.85	64.10	61.54	66.67	69.23	84.62
**28**	**79.49**	**61.54**	**61.54**	**61.54**	**61.54**	**61.54**	**61.54**	**64.10**	**69.23**	**92.31**

Each model’s performance was represented as a test accuracy. The row that has the highest test accuracy and corresponding number of the feature were marked to bold.

**TABLE 4:** Information of 28 features used in a AdaboostM1 model.

Feature	Power	Band	Channel	Feature	Power	Band	Channel
1	Abs	Delta	Fp1	15	Rel_zscore	Delta	T3
2	Abs	Alpha1	Fp1	16	Rel_zscore	Delta	C3
3	Abs	Alpha1	F7	17	Rel_zscore	Delta	C4
4	Abs	Beta1	O2	18	Rel_zscore	Theta	T4
5	Abs	Beta3	F7	19	Rel_zscore	Alpha1	F3
6	Abs	Beta3	P3	20	Rel_zscore	Alpha1	T5
7	Rel	Delta	T6	21	Rel_zscore	Alpha1	O2
8	Rel	Beta1	P4	22	Rel_zscore	Alpha2	Fp1
9	Rel	Beta2	C3	23	Rel_zscore	Alpha2	P3
10	Rel	Beta3	F4	24	Rel_zscore	Alpha2	Pz
11	Abs_zscore	Delta	Pz	25	Rel_zscore	Beta1	C4
12	Abs_zscore	Theta	T6	26	Rel_zscore	Beta2	C3
13	Abs_zscore	Beta2	T6	27	Rel_zscore	Beta2	T5
14	Rel_zscore	Delta	F7	28	Rel_zscore	Beta3	T3

Abs and Rel represents Absolute band power and Relative band power, respectively. Abs_zscore and Rel_zscore represents Absolute z-scored band power and Relative z-scored band power, respectively.

## Discussion

The variation of QEEG in individuals by age and sex can disturb finding disease-specific biomarkers. To prevent this, the sex and age-matched standardized feature were extracted through Norm-DB in the present study. The corrected features helped to remove irrelevant effects on disease-specific features. The final features were selected by a common top-ranked feature importance value in a tree-based ensemble model. Feature importance is scored according to how much each feature influenced the learning and prediction results of the model. In consequence, the number of sex and age-matched standardized features was overwhelming among the top 28 finally selected features. Therefore, it proves that features, considering age and gender, outperformed compared to original features.

The present study aims to provide an auxiliary diagnostic tool that aids in the early screening of potential depression, not a replacement of the current clinical diagnostic criteria. Through our prediction model, clinical communities may efficiently diagnose and distinguish the patients who are potentially depressed and in need of pre-emptive treatment. In addition, EEG-based early screening is much easier to access through clinical experts without professionals. Consequently, it may eventually enable the conduction of early screenings at home by individuals, as digital mental health care expands in our society.

By all means, it is difficult to deem BDI score a complete replacement for a diagnosis of depression. The result of intergroup comparison classified based on BDI scores may be incomplete compared to results between groups classified based on the diagnoses of the specialist. However, BDI surveys provide specific questionnaires based on patients’ experience over the past 4 weeks, hence, it can exclude the case that symptom appears temporally. There are also many previous studies that proved the reliability of BDI with comparison to specialists’ diagnoses (19–21).

Our group analysis results showed which electrode and band power can be biomarkers of depression, which are consistent with the results based on clinical diagnoses of prior studies (6, 22). The strength of our study is that our model can discern patients with potential depression using quantitative biomarkers, such as high beta and low alpha2. This suggests that patients with potential depression can realize and pre-emptively respond to their states, even though they are in an insufficient environment (lack of time, no specialists, etc.).

To overcome the limitation, an accurate intergroup comparison with data labeled by a specialist will be made in a further study. Discovering biomarkers for accompanied diseases with depression, such as anxiety and bipolar affective disorder, or phenotype of depression will also be made. In addition, the application of other features, such as alpha asymmetry or source-level features will be considered (23, 24).

Nevertheless, our study discovered and provided a distinct biomarker for patients with depression through a unique method of considering the gender and age of subjects. The results of this study are believed to greatly contribute to the further study of digital mental healthcare and clinical facilities.

## Data availability statement

The data analyzed in this study is subject to the following licenses/restrictions: This data is the property of iMediSync Inc., and can be provided on a reasonable request. Requests to access these datasets should be directed to www.imedisync.com.

## Ethics statement

The studies involving human participants were reviewed and approved by the Research Ethics Committee of the Seoul National University. The patients/participants provided their written informed consent to participate in this study.

## Author contributions

TK did overall works from analysis to modeling and also wrote the manuscript. UP assisted TK. SK gave some comments about the project. All authors contributed to the article and approved the submitted version.

## Footnotes

1
https://isyncbrain.com/
